# Treatment of acute basilar artery occlusion by retrograde puncture via the vertebral artery approach: A care-compliant case report

**DOI:** 10.1097/MD.0000000000038617

**Published:** 2024-06-21

**Authors:** Zi Wen Wang, Fang Zhao, Jin Chao Liu

**Affiliations:** a Radiology Intervention Department, Puyang Oilfield General Hospital, China.

**Keywords:** basilar artery, mechanical thrombectomy, occlusion, retrograde puncture

## Abstract

**Background:**

Vertebral artery stump syndrome (VASS) is a cause of acute stroke. Owing to the particularity of the pathogenesis of VASS, interventional treatment of VASS is difficult. Common mechanical thrombectomy approaches include femoral and radial artery approaches. However, conventional approaches may not be suitable for VASS. If effective measures are not taken to open offending vessels in time, this can lead to a high rate of disability. In recent years, no consensus has been reached regarding surgical methods for treating VASS.

**Patient concerns:**

The patient presented to the emergency department with a 2-hour history of disturbance of consciousness.

**Diagnosis:**

After neurological and magnetic resonance imaging examinations, the patient was diagnosed with acute large vessel occlusive posterior circulation cerebral infarction.

**Methods:**

The patient’s symptoms were not relieved after intravenous infusion of argatroban (10 mg) at a local hospital. We first attempted to open the occluded vertebral artery through normal approaches but failed. We then punctured the vertebral artery, successfully opened the occluded vertebral artery, and performed mechanical thrombectomy.

**Results:**

The patient underwent successful vertebral artery puncture and mechanical thrombectomy, with no evidence of postoperative bleeding or vascular injury at the puncture site. The patient regained consciousness the day after surgery but remained impaired in physical activity. After 4 months of rehabilitation, the patient recovered completely.

**Conclusion:**

When the conventional approach cannot meet the requirements of mechanical thrombectomy, reverse puncture of the vertebral artery is a feasible surgical method for patients with VASS. However, due to the small number of cases, a series of safety problems such as potential puncture failure, hemorrhage after puncture, and vascular occlusion still need to be further explored.

## 1. Introduction

Common mechanical thrombectomy approaches include the femoral and radial artery approaches. However, conventional approaches may not be suitable for treating vertebral artery stump syndrome (VASS), where there is no obvious stump near the cardiac end of the occluded vessel of the occluded segment. At present, a few studies^[1–3]^ have reported that direct vertebral artery puncture and mechanical thrombectomy have successfully restored cerebral perfusion, and no studies have reported any attempt to open the original diseased vessels, which may be potentially unsafe and lead to thromboembolism recurrence. Here, we report a case of VASS. We punctured the vertebral artery, successfully opened the occluded vertebral artery, and performed mechanical thrombectomy.

## 2. Case presentation

A 68-year-old female presented to the emergency department with a 2-hour history of disturbance of consciousness. When the patient was found unconscious by the roadside, she was in a state of shallow coma; thus, the specific onset time is unknown, and when the patient’s symptoms worsened is also unclear. The patient had a history of hypertension and coronary heart disease. Upon arrival at the emergency department, the patient’s systolic blood pressure was measured at 200 mm Hg.

### 2.1. Diagnosis

**Figure 1.**
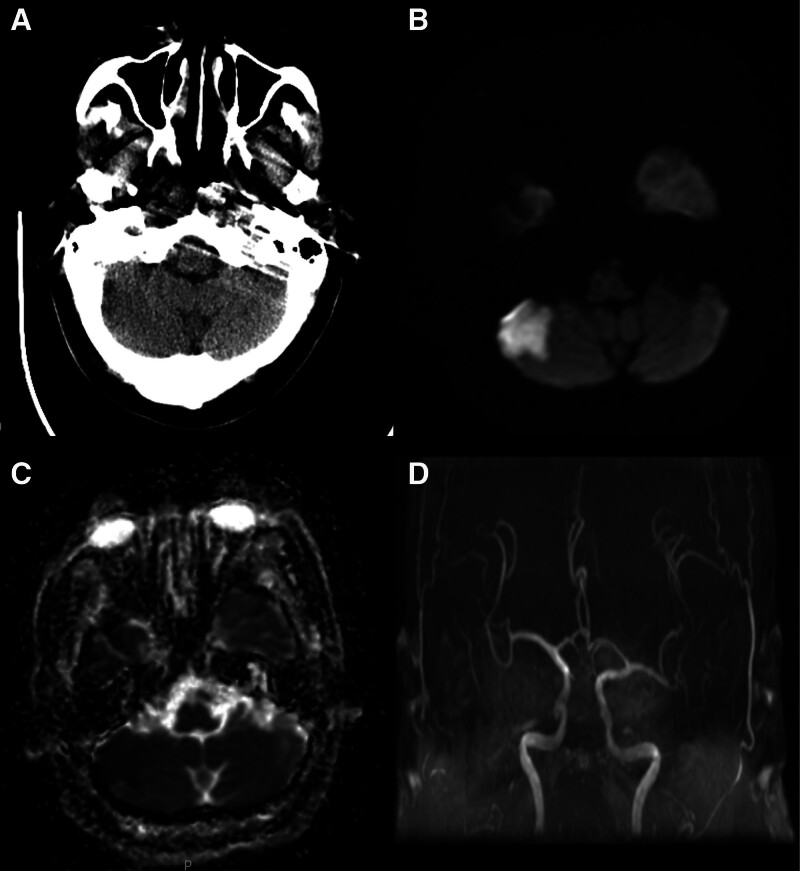
(A) Cranial computed tomography (CT) showed no evidence of intracranial hemorrhage. (B and C) Diffusion-weighted imaging and apparent diffusion coefficient images indicating acute infarcts in the right pons and cerebellar hemisphere. (D) Cranial magnetic resonance angiography (MRA) image indicating alterations suggestive of basilar artery occlusion.

Cranial magnetic resonance imaging (MRI) conducted at the local hospital revealed a complete occlusion of the basilar artery. Diffusion-weighted imaging and apparent diffusion coefficient images indicated acute infarcts in the right pons and cerebellar hemisphere. Cranial computed tomography (CT) revealed no evidence of intracranial hemorrhage (Fig. 1). Based on the comprehensive neurological examination and imaging findings, the patient in this case was diagnosed with acute basal artery occlusion. Since our hospital was unable to perform high-resolution MRI, the specific cause of occlusion required further refinement through cerebral angiography evaluation and the development of a treatment plan.

### 2.2. Therapeutic intervention

**Figure 2.**
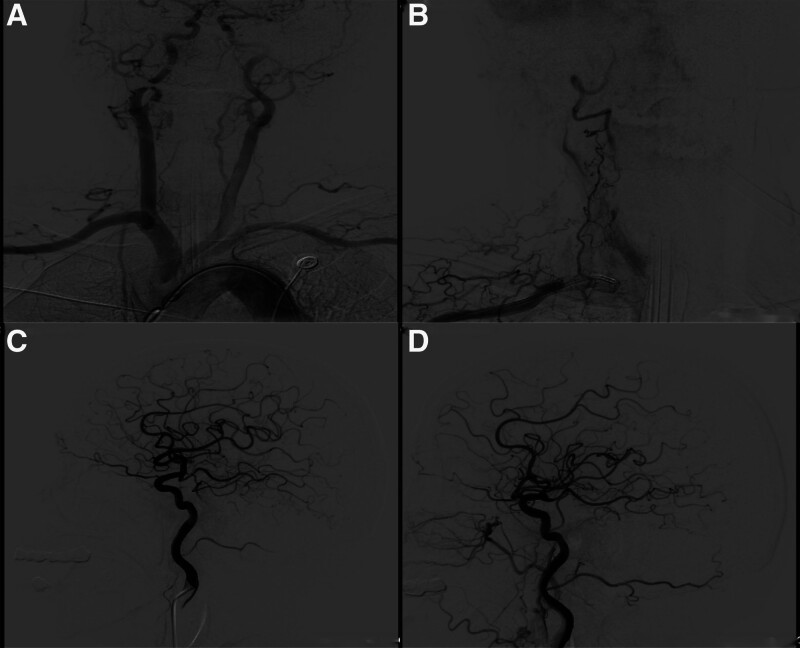
(A) Cerebral angiography showed that the right vertebral artery was the dominant vertebral artery and that its opening was completely occluded. The left vertebral artery is slender and does not converge to the basilar artery. (B) The blood supply was compensated for by the branches of the thyroid neck trunk ascending carotid artery at the distance of the V2 segment, and the distal part of the basilar artery was completely occluded. (C and D) Bilateral internal carotid angiography showed that the bilateral posterior cerebral arteries were embryonic posterior cerebral arteries, and the posterior communicating arteries were not opened.

**Figure 3.**
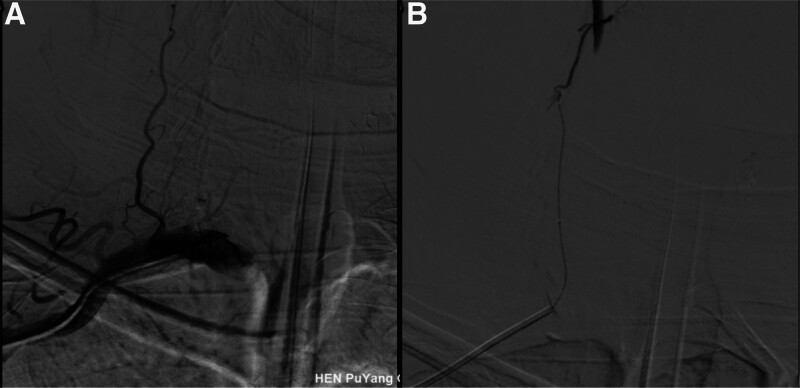
(A) We first attempted to open the occluded vertebral artery through the femoral artery approach and the radial artery approach. (B) We did not successfully open the vertebral artery through the collateral vessels (SHERPA technique).

**Figure 4.**
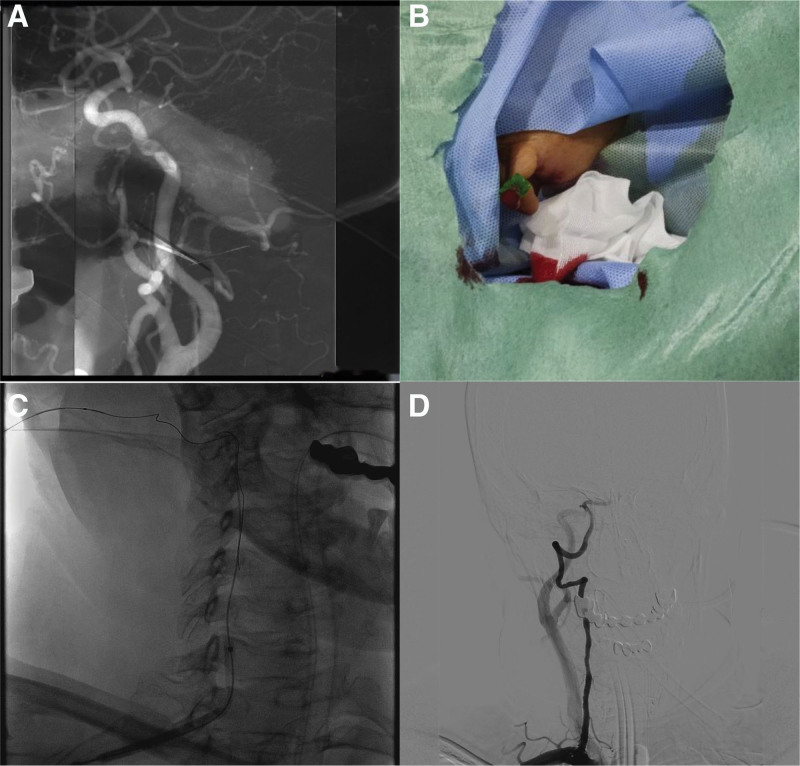
(A) The V3 segment of the vertebral artery was punctured under the guidance of the road map. (B) After successful puncture, arterial blood can be observed protruding from the puncture needle. (C) By tracing the micro guide wire, we successfully located the opening of the vertebral artery. (D) After balloon dilation and stent implantation, the initial segment of the vertebral artery was reopened, and the blood was restored.

The patients’ symptoms were not relieved by intravenous argatroban infusion (10 mg) at a local hospital. Thrombolysis may increase the risk of intracranial hemorrhage. After fully informing the patient’s family of the risks and benefits of intravenous thrombolysis, they declined the treatment and requested endovascular therapy. We used a conventional approach (the right radial artery) for cerebral angiography. The results showed that the right dominant vertebral artery was completely occluded at its opening (Fig. 2A). The blood supply was compensated by the branches of the thyroid neck trunk ascending vertebral artery at the distance of the V2 segment, and the distal part of the basilar artery was completely occluded (Fig. 2B). The left vertebral artery was fully occluded in the posterior inferior cerebellar artery. Bilateral internal carotid angiography revealed that the bilateral posterior cerebral arteries were embryonic posterior cerebral arteries (Fig. 2C and D). The National Institutes of NIHSS score at admission was 33 points. Given the DSA showing occlusion of the dominant side of the vertebral artery, the filling of collateral vessels in the V2 segment of the vertebral artery, and the occlusion of the basilar artery, the etiology of this stroke is considered acute thrombosis within the vertebral artery on the basis of chronic vertebral artery occlusion or embolism from a thrombus detached from the vertebral artery occluding the basilar artery. Considering the patient’s clinical symptoms and the supplemented imaging data, the possibility of hemodynamic stroke can be excluded. We first attempted to open the occluded vertebral artery through the femoral and radial artery approaches (Fig. 3A). Neither approach successfully opened the vertebral artery through the collateral vessels (SHERPA technique^[4]^) (Fig. 3B). We then punctured the V3 segment of the vertebral artery under the guidance of the road map (Fig. 4A and B) and introduced a microguidewire to probe the occluded segment at the beginning of the vertebral artery. We successfully located the opening of the right vertebral artery under the tracing effect of the microguidewire (Fig. 4C). After removing the microguidewire, local compression was applied for hemostasis, with a compression time of 5 minutes. Repeat angiography performed 5 minutes later revealed no extravasation of contrast agent at the puncture site, suggesting closure of the puncture point. The catheter and guidewire were introduced through the right radial artery approach to successfully open the occluded segment of the vertebral artery. Balloon dilatation, catheter thrombus extraction, and stent implantation were performed at the vertebral artery opening (Fig. 4D). A Catalyst 5 (Stryker Corporation) intermediate catheter was utilized to perform ADAPT thrombectomy by approaching the occluded segment of the basilar artery. After thrombus aspiration, stenotic lesions in the basilar artery were revealed by angiography. Regrettably, due to oversight during the thrombus aspiration procedure, images were not retained.

### 2.3. Follow-up and outcomes

**Figure 5.**
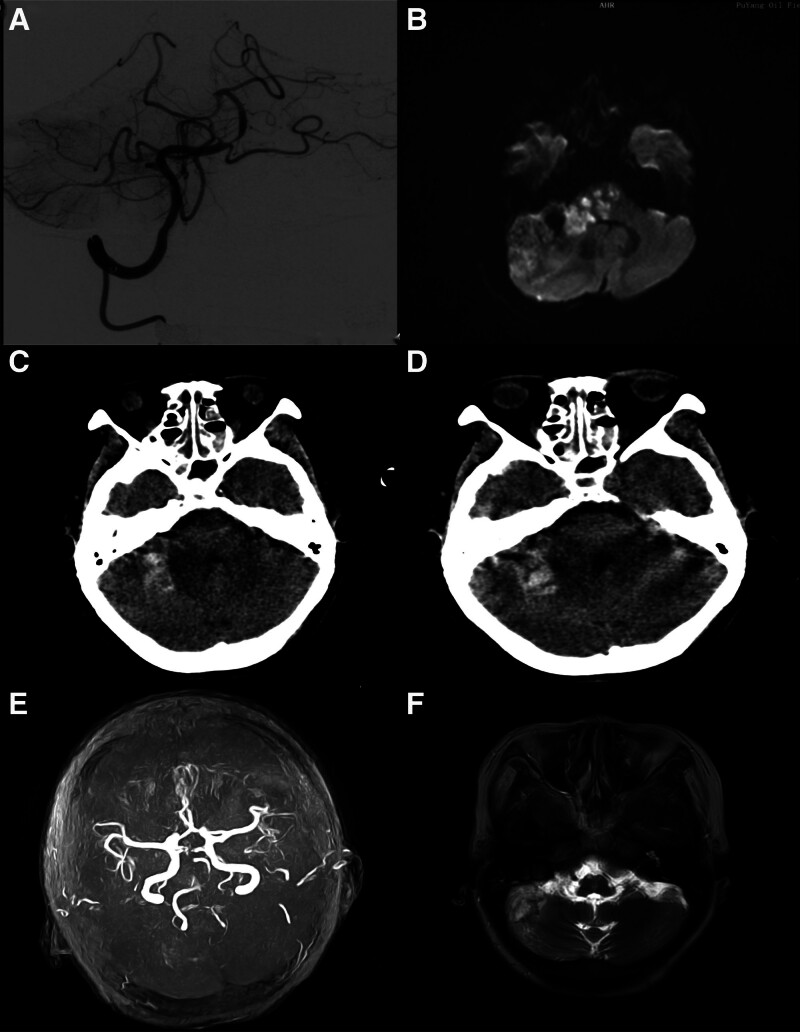
(A) Blood flow in the basilar artery was restored after mechanical thrombectomy (mTICI III). (B) MRI of the patient at 48 h after the operation showing punctate hemorrhage in the right cerebellar hemisphere, pons and pontine arm with multiple infarcts. (C and D) Cranial CT image indicating high-density signs in the right cerebellar hemisphere and pons, suggestive of localized hemorrhagic changes. (E) MRA image indicating patency of the basilar artery lumen at 4 months after surgery. (F) MRI examination revealed no new infarcts after 4 months of follow-up.

The patient underwent successful vertebral artery puncture and mechanical thrombectomy, with no evidence of postoperative bleeding or vascular injury at the puncture site. Blood flow was restored after mechanical thrombectomy of the basilar artery (mTICI III) (Fig. 5A). MRI and CT of the patient at 48 h after the operation revealed punctate hemorrhage in the right cerebellar hemisphere, pons, and pontine arm with multiple infarcts (Fig. 5B–D), and the NIHSS score decreased to 24 points. After subsequent rehabilitation and drug treatment, we followed up again at 4 months, and the patient recovered and took care of himself. The NIHSS score was measured on a 3-point scale. Magnetic resonance imaging (MRI) revealed no new infarcts (Fig. 5E and F).

## 3. Discussion

Recently, endovascular therapy (EVT) has been considered a promising treatment option for acute basilar artery occlusion (ABAO),^[5]^ as standard medical therapy alone is characterized by insufficient recanalization rates and poor prognosis in patients with ABAO.^[6]^ As a special disease type, VASS is difficult to treat because of the particularity of complete occlusion of the initial part. Intravenous thrombolysis is an effective method for treating acute cerebral infarction within 6 hours. The exact onset time of the patient’s condition is unknown. Therefore, the thrombolysis time window may have been exceeded. Ji et al^[7]^ and Gross et al^[4]^ reported cases of retrograde opening of the occluded vertebral artery through collateral vessels to the main vertebral artery. We also attempted this during the operation, but the microcatheter and guidewire could not be passed through. Liu et al^[8]^ reported mechanical thrombectomy via the anterior and posterior communicating arteries to the basilar artery. Because the anterior and posterior communicating arteries of the patient were not connected to the basilar artery, this method could not be implemented.

Moniz was the first to describe direct vertebral artery puncture in the 1920s.^[9]^ Later, Semeraro et al^[2]^ and O’Reilly et al^[1]^ reported that the V2 and V3 segments of the vertebral artery were punctured under color Doppler ultrasound guidance for direct mechanical thrombectomy. Elhorany et al^[10]^ and Nawabi et al^[3]^ reported that the V2 and V3 segments of the vertebral artery were punctured under the guidance of a road map for mechanical thrombectomy. The above case reports directly describe the success of mechanical thrombectomy through the puncture point approach for basilar artery occlusion, and there is no need for further treatment of vertebral artery stenosis or residual thrombosis, which can increase the risk of re-embolism. After vertebral artery puncture, we first reverse-opened the occluded vertebral artery and aspirated the thrombus, which greatly reduced the risk of stroke. Regarding hemostasis treatment at the puncture point, Semeraro et al^[2]^ and Elhorany et al^[10]^ reported that manual compression and vascular plugs were used to stop bleeding at the puncture point after the operation, which may lead to complications such as vertebral artery stenosis and thromboembolism at the puncture point. During the operation, we implanted a guide wire only after successful puncture of the vertebral artery, which reduced puncture injury to the vertebral artery, and manual compression was not needed after the operation, which increased the lumen diameter of the vertebral artery and posterior circulation blood flow. Additionally, due to the small diameter of the vertebral artery, none of the vascular closure devices currently available on the market are indicated for closure of the vertebral artery. Therefore, we did not attempt closure.

In conclusion, when the conventional approach cannot meet the requirements of mechanical thrombectomy, the reverse puncture technique for the vertebral artery is a feasible surgical method for patients with VASS. However, due to the small number of cases, a series of safety problems such as potential puncture failure, hemorrhage after puncture, and vascular occlusion still need to be further explored.

## Acknowledgments

The authors thank AJE (www.aje.cn) for English language editing.

## Author contributions

**Writing – original draft:** Ziwen Wang.

**Visualization:** Fang Zhao.

**Writing – review & editing:** Jin Chao Liu.
